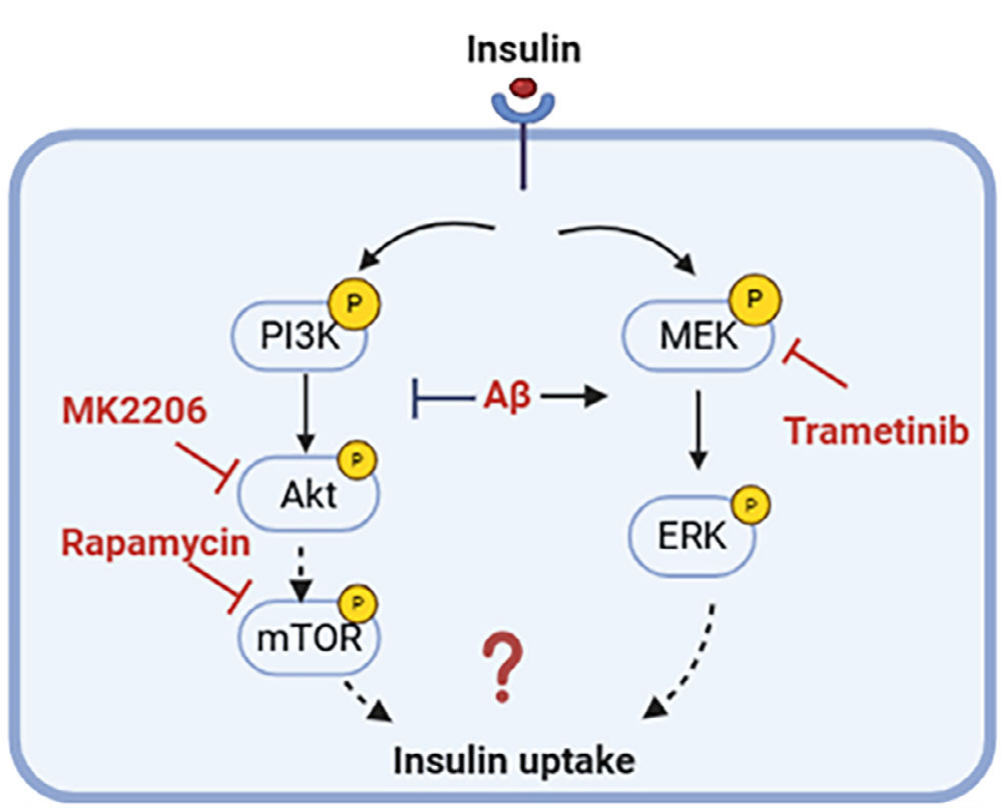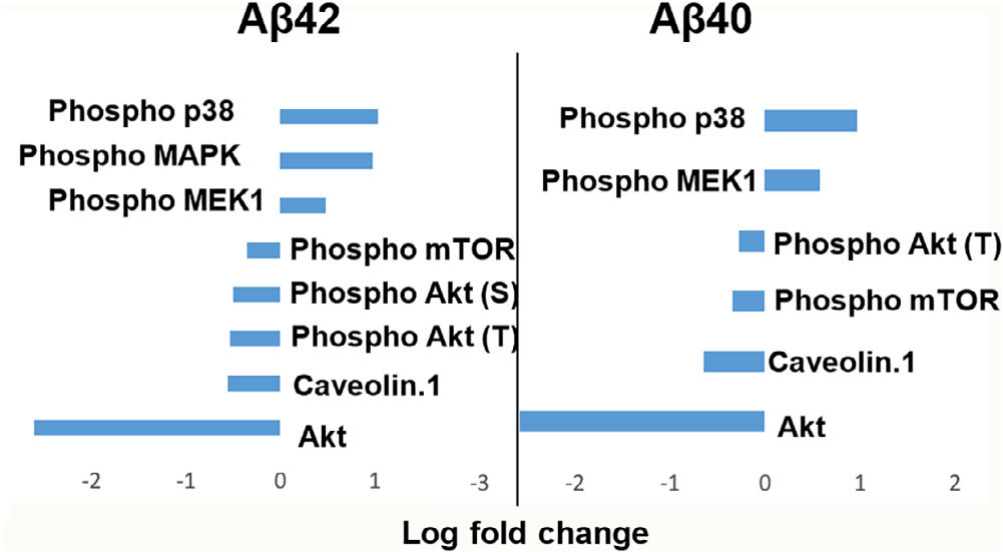# Amyloid beta peptides inhibit insulin signaling and trafficking at the blood‐brain barrier in Alzheimer's disease

**DOI:** 10.1002/alz70861_109020

**Published:** 2025-12-23

**Authors:** Vaishnavi Veerareddy

**Affiliations:** ^1^ University of Minnesota, Minneapolis, MN USA

## Abstract

**Background:**

Alzheimer’s disease (AD) is a progressive neurodegenerative disease characterized by cognitive decline, amyloid plaques, and hyperphosphorylated tau. It is widely believed that metabolic syndrome, which results in endothelial insulin resistance, aggravates AD risk and accelerates cognitive decline by disrupting insulin signaling and reducing brain insulin levels. Given the cardinal role of insulin in maintaining neurotrophic functions, several strategies were attempted to restore brain insulin levels in the AD brain. However, they did not yield expected results, most likely due to off‐target effects. Therefore, it is critical to elucidate pathophysiological mechanisms driving insulin resistance and impaired insulin delivery to the brain. We hypothesize that soluble amyloid beta (Aβ) peptides accumulating in the Alzheimer’s brain inhibit insulin signaling and trafficking at the BBB and trigger brain insulin resistance.

**Method:**

Brain insulin uptake was assessed in APP/PS1 mice and their non‐transgenic littermates. The effect of Aβ peptides on the insulin signaling pathway was investigated using reverse‐phase protein array (RPPA) methods in the blood‐brain barrier (BBB) cell culture model. To examine the impact of Aβ exposure, wild‐type mice were pre‐infused with Aβ peptides, and changes in insulin signaling and trafficking at the BBB were assessed. Mechanisms of insulin resistance upon Aβ exposure were elucidated in the BBB cell culture models using specific insulin signaling inhibitors.

**Result:**

The Aβ exposure decreased the brain insulin uptake rate in vivo. Exposure to Aβ40, a vasculotropic peptide, increased insulin binding to the insulin receptor while decreasing insulin uptake, potentially through uncompetitive inhibition. A disruption in insulin signaling accompanies this observed reduction in insulin uptake. The RPPA analysis has demonstrated that Aβ peptides differentially disrupt the metabolic (PI3K/AKT/mTOR) and mitogenic (MEK/ERK) arms of the insulin signaling pathway. Treatment with MK2206 (AKT inhibitor) decreased insulin uptake, thus implicating AKT activation in insulin uptake. Conversely, the treatment of trametinib, a MEK inhibitor, did not affect insulin uptake.

**Conclusion:**

Aβ peptides decrease the brain insulin delivery via the BBB by inhibiting the PI3K/AKT pathway.